# Expression and characterization of recombinant Par j 1 and Par j 2 resembling the allergenic epitopes of *Parietaria judaica* pollen

**DOI:** 10.1038/s41598-019-50854-1

**Published:** 2019-10-21

**Authors:** Yulia Dorofeeva, Paolo Colombo, Miguel Blanca, Adriano Mari, Roman Khanferyan, Rudolf Valenta, Margarete Focke-Tejkl

**Affiliations:** 1 Division of Immunopathology, Department of Pathophysiology and Allergy Research, Center of Pathophysiology, Infectiology and Immunology, Medical University of Vienna, Vienna, Austria; 2 Istituto di Biomedicina ed Immunologia Molecolare “Alberto Monroy” del Consiglio Nazionale delle Ricerche, Palermo, Italy; 3 Hospital Civil, Malaga, Spain; 4 Associated Centers for Molecular Allergology, Rome, Italy; 5 Russian People’s Friendship University, Moscow, Russian Federation; 6 The Institute of Immunology, Moscow, Russian Federation; 7 Laboratory for Immunopathology, Department of Clinical Immunology and Allergy, Sechenov First Moscow State Medical University, Moscow, Russian Federation

**Keywords:** Immunology, Inflammation

## Abstract

The weed wall pellitory, *Parietaria judaica*, is one the most important pollen allergen sources in the Mediterranean area causing severe symptoms of hay fever and asthma in allergic patients. We report the expression of the major *Parietaria* allergens, Par j 1 and Par j 2 which belong to the family of lipid transfer proteins, in insect cells. According to circular dichroism analysis and gel filtration, the purified allergens represented folded and monomeric proteins. Insect cell-expressed, folded Par j 2 exhibited higher IgE binding capacity and more than 100-fold higher allergenic activity than unfolded *Escherichia coli*-expressed Par j 2 as demonstrated by IgE ELISA and basophil activation testing. IgE ELISA inhibition assays showed that Par j 1 and Par j 2, contain genuine and cross-reactive IgE epitopes. IgG antibodies induced by immunization with Par j 2 inhibited binding of allergic patients IgE to Par j 1 only partially. IgE inhibition experiments demonstrated that insect cell-expressed Par j 1 and Par j 2 together resembled the majority of allergenic epitopes of the *Parietaria* allergome and therefore both should be used for molecular diagnosis and the design of vaccines for allergen-specific immunotherapy of *Parietaria* allergy.

## Introduction

IgE-associated allergy is the most common hypersensitivity disease affecting more than 25% of the world´s population^1,2^. Mainly in the Mediterranean area but also at the southern coast of UK, France, Australia, California and Eastern Europe the weed *Parietaria judaica*, wall pellitory, is one the most common pollen allergen sources^3–7^. *Parietaria judaica* pollen is responsible for severe forms of respiratory allergy (i.e., severe allergic rhinitis, asthma) which may be due to the fact that the plant has an unusually long period of pollination ranging from February to November^8–10^. Already an early report from 1956 identified *Parietaria* as important allergen source responsible for allergic asthma, one of the most severe manifestations of allergy^11^. Approximately 40 years later the molecular nature of the two most important allergens from *Parietaria*, Par j 1 and Par j 2 was revealed by cDNA cloning techniques^12,13^. Par j 1 and Par j 2 are non-glycosylated allergens with a molecular weight of 15 kDa and 11.3 kDa respectively. They belong to the family of lipid transfer proteins (LTP) which typically consist of four alpha-helices that are linked and stabilized by four disulphide bonds^14–17^. In addition to Par j 1 and Par j 2, highly cross-reactive allergens belonging to the family of profilins and calcium-binding allergens are minor allergens in *Parietaria* which are recognized typically by less than 10% of the patients^18,19^. The expression and purification of recombinant Par j 1 and Par j 2 resembling the fold of the corresponding wild-type allergens is difficult and requires eukaryotic hosts such as yeast expression systems capable of forming correct disulphide bonds^20^. In fact, it was reported that yeast-expressed Par j 1 and Par j 2 showed a similar fold as the natural allergens. Yeast-expressed Par j 1 and Par j 2 were recognized by sera from *Parietaria*-allergic patients and exhibited allergenic activity as demonstrated by skin testing^20^. Accordingly the recombinant allergens were suggested to be useful for the diagnosis of allergy to *Parietaria* by component-resolved diagnosis. In fact, molecular allergy diagnosis offers important advantages over conventional allergen-extract-based diagnosis because it allows revealing the allergic patients molecular sensitization pattern and thus aids in the diagnostic resolution of difficult cases and in the refined prescription of allergen-specific immunotherapy^21–23^, the only causal and disease-modifying treatment for IgE-associated allergies^24^. In the meantime, molecular approaches for allergen-specific immunotherapy have been successfully evaluated in many clinical trials and hold promise to improve AIT in the future^25,26^. In the case of *Parietaria* allergy several approaches have been considered^27–29^ but the question remains what allergen molecules need to be included in the vaccine. Regarding Par j 1 and Par j 2 it is known that the allergens contain cross-reactive epitopes^30^ but the open question is if, due to cross-reactivity, one can replace the other allergen. Furthermore there are no detailed studies investigating the allergenic activity of the two allergens. Finally and importantly, it has not yet been determined what allergens can resemble the spectrum of allergenic epitopes of the *Parietaria* allergome to replace allergen extract-based vaccines.

In order to address these open questions we have expressed folded rPar j 1 and rPar j 2 in insect cells and studied the allergenic activity of both recombinant allergens by titrated basophil activation testing. Furthermore we studied the extent of IgE cross-reactivity of rPar j 1 and rPar j 2 by IgE inhibition studies and demonstrated that only both allergens together resemble the spectrum of IgE epitopes of natural *Parietaria* pollen extract.

## Results

### Physicochemical characterization of purified recombinant Par j 2 and Par j 1

**Figure 1:**
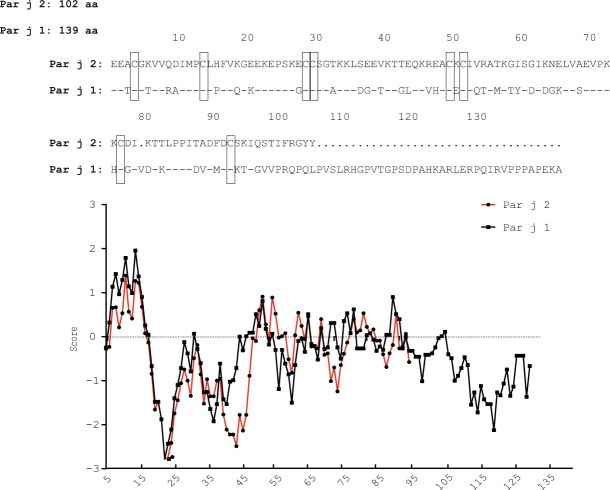
Alignment of the amino acid sequences of Par j 2 and Par j 1 (top) and hydrophobicity plots (bottom). Identical amino acids are indicated by dashes, dots indicate gaps and conserved cysteine residues are boxed.

Figure 1a shows an alignment of the deduced amino acid sequences of Par j 2 (P55958) and Par j 1 (O04404). In the overlapping region the proteins show a 53% sequence identity. Par j 1 contains additional 37 amino acids at its C-terminal end. The hydrophobicity prediction performed with the Kyte and Doolittle algorithm shows that the proteins contain a highly hydrophobic N-terminus followed by a hydrophilic stretch and a region with intermediate hydrophobicity. The C-terminus of Par j 1 shows high hydrophilicity. Par j 1 and Par j 2 contain eight conserved cysteine residues (Fig. 1, boxed). Supplementary Fig. S1a displays the sequence identities of Par j 2 and Par j 1 with LTPs identified as allergens in other plants and in plant tissues other than pollen. Both, Par j 2 and Par j 1, showed a relatively low sequence identity of less than 35% with the other plant LTP allergens whereas sequence identities of up to >90% (e.g., Pru p 1 vs. Pru ar 3) between certain LTPs, mainly from somatic tissues (i.e., plant food) were found (Supplementary Fig. S1a, colors). Accordingly, the phylogenetical comparison analysis of the LTPs shows that Par j 1 and Par j 2 represent an independent branch highlighting the evolutionary differences with other proteins of this family as represented in Supplementary Fig. S1b. However, it should be noted that neither the degree of sequence identities nor the phylogenetic relationships of the different LTPs were associated with the expression in certain plant tissues (pollen versus somatic tissues) or with the botanical relationships of the corresponding plants (monocotyledonic versus dicotyledonic plants).

**Figure 2:**
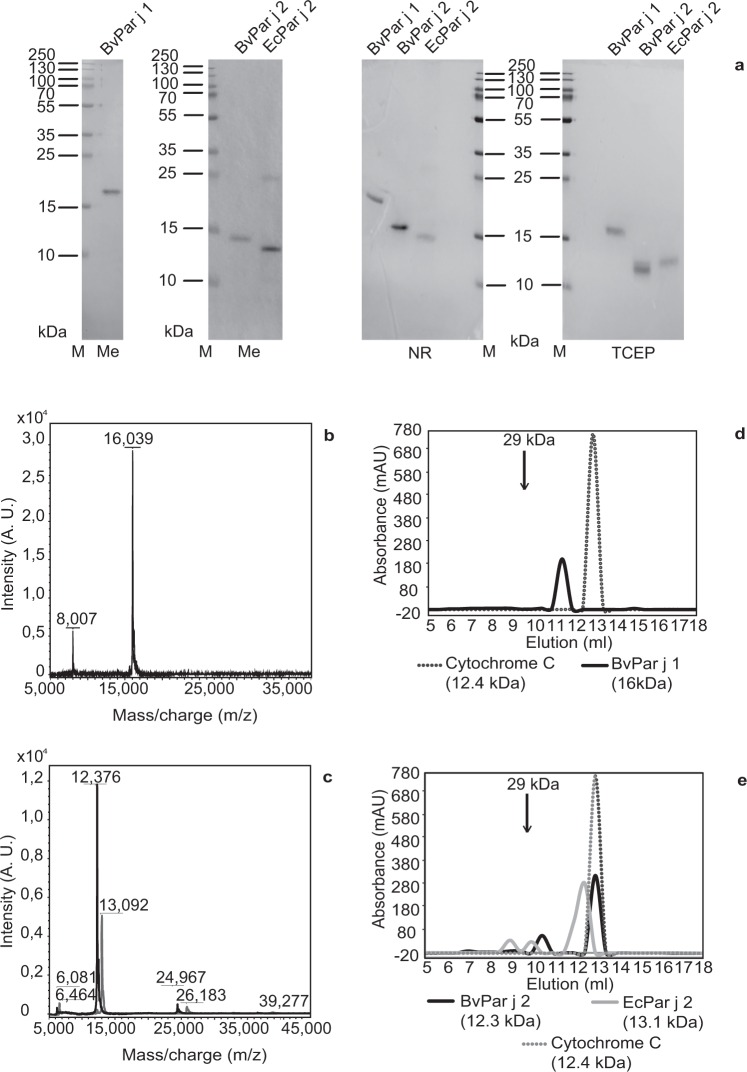
Biochemical and biophysical characterization of purified BvPar j 1, BvPar j 2 and EcPar j 2. Coomassie brilliant blue-stained SDS-PAGE containing purified BvPar j 1, BvPar j 2 and EcPar j 2 (**a**) separated under non-reducing conditions (panel NR), reducing conditions using β-Mercaptoethanol (panel Me) or Tris(2-carboxyethyl)phosphine (panel TCEP). Molecular weight markers are indicated on the left margins in kilo Dalton (kDa). Full-length gels are presented in Supplementary Fig. S1. Matrix-assisted laser desorption and ionization-time-of-flight (MALDI-Tof) analysis of (**b**) BvPar j 1, (**c**) BvPar j 2 (black) and EcPar j 2 (grey). The mass/charge ratios are indicated on the x-axes, and the intensities are shown as absorption units of the most intensive signals obtained in the mass range (y-axes). Analysis of (**d**) BvPar j 1, (**e**) BvPar j 2 (black) and EcPar j 2 (grey) by gel filtration. Cytochrome C (dotted peak) and carbonic anhydrase (indicated by an arrow at 29 kDa) served as molecular weight standards. Elution volumes are shown in *milliliters -* mL (x-axes) and the absorbances of the proteins at 280 nm are indicated in *milli-absorbance* units - mAU (y-axes).

We expressed recombinant Par j 1 and Par j 2 proteins in baculovirus-infected insect cells and named them BvPar j 1 and BvPar j 2, respectively. Both proteins were compared with recombinant Par j 2 expressed in *E. coli* (EcPar j 2)^12^. Recombinant proteins were purified by Ni^2+^-affinity chromatography to homogeneity. Purified proteins were then analysed by SDS-PAGE under reducing and non-reducing conditions (Fig. 2a). BvPar j 2 migrated as single band of approximately 15 kDa under non-reducing conditions and as 14 kDa band under reducing conditions using ß-Mercaptoethanol. Under non-reducing conditions EcPar j 2 showed a main band of approximately 14 kDa and an additional band of 27 kDa and appeared as 13 kDa band when using ß-Mercaptoethanol. BvPar j 1 migrated as a monomeric band of approximately 18 kDa under non-reducing and as 17 kDa under reducing conditions (ß-Mercaptoethanol) (Fig. 2a).

In SDS-PAGE both insect cell-expressed proteins appeared to have greater masses than their calculated masses (BvPar j 2: 12,380 kDa; BvPar j 1: 16,020 kDa) whereas the mass of the bacterially expressed EcPar j 2 protein corresponded to the predicted mass (i.e., 12,744 kDa) under reducing and non-reducing conditions and a band of approximately 26 kDa likely to be a dimer. Taking into account that the structures of LTPs are stabilized by 4 disulphide bonds the discrepancy in masses might be explained by retained fold of the proteins even under denaturing conditions. We therefore performed SDS PAGE using Tris(2-carboxyethyl)phosphine (TCEP), a reagent which is more potent in reducing disulphide bonds than ß-Mercaptoethanol (Fig. 2a., right part). When TCEP was used EcPar j 2 appeared as a monomeric band of predicted mass and now BvPar j 1 and BvPar j 2 exhibited masses which corresponded to those which had been calculated (Fig. 2a).

Next, the purified proteins were analysed by MALDI mass spectrometry where BvPar j 1 showed a peak of 16,039 kDa (Fig. 2b) corresponding to the mass calculated from the amino acid sequence (16,020 kDa).

When BvPar j 2 and EcPar j 2 were checked by mass spectrometry, both showed high intensity peaks with masses of 12,376 kDa and 13,092 kDa, respectively (Fig. 2c). The measured mass of BvPar j 2 fitted the calculated mass of 12,380 kDa whereas the mass measured for EcPar j 2 was higher than the expected mass of 12,744 kDa which may be attributed to the presence of an N-terminal methionine. When comparing BvPar j 2 and EcPar j 2 in size exclusion experiments by gel filtration both insect cell- and *E. coli*-expressed proteins appeared at about the same elution volume as the standard Cytochrome C (EcPar j 2 > BvPar j 2) (12.4 kDa) (Fig. 2e). BvPar j 2 contained also a small dimer peak of approximately 25 kDa and EcPar j 2 showed the presence of dimers and trimers of approximately 26 kDa and 39 kDa, respectively (Fig. 2e). BvPar j 1 occurred as a strictly monomeric form confirming the data obtained by mass spectrometry (Fig. 2b).

**Figure 3:**
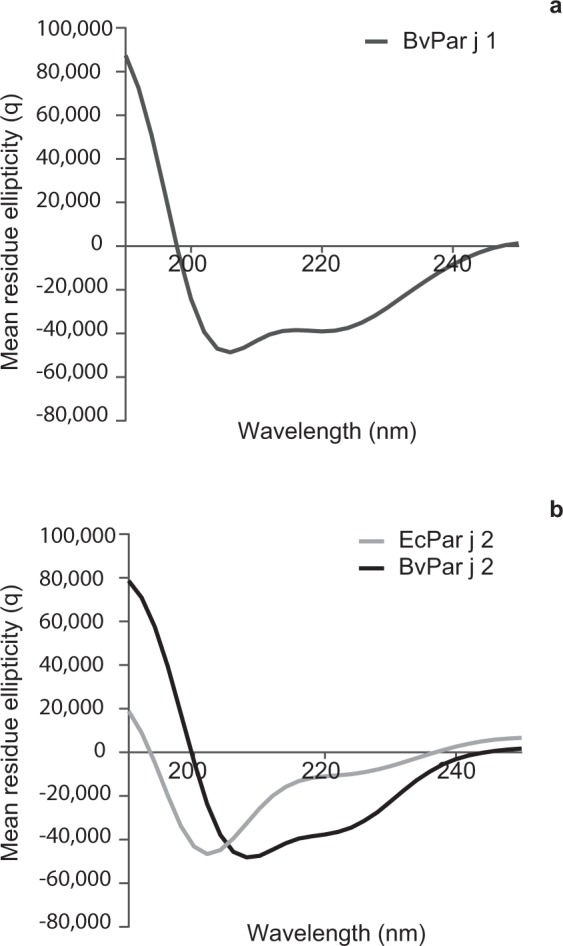
Far-UV circular dichroism analysis of (**a**) BvPar j 1, (**b**) BvPar j 2 (black) and EcPar j 2 (grey). Shown are mean residue ellipticities Θ (y-axes) at the given wavelengths (x-axes).

We then compared the secondary structure of the proteins expressed in insect cells and *E. coli* by analysis of their far-ultraviolet circular dichroism (far-UV CD) spectra (Fig. 3). The spectra of BvPar j 1 (Fig. 3a) and BvPar j 2 (Fig. 3b) showed almost the same curves resembling α-helical structures with theoretical minima at 208 nm, a defined shoulder at 221 nm, and a maximum at 190 nm whereas EcPar j 2 showed an unordered conformation (Fig. 3b).

### Insect cell-expressed Par j 2 shows significantly higher IgE reactivity and allergenic activity than *E. coli*-expressed Par j 2: Importance of conformational IgE epitopes

**Figure 4:**
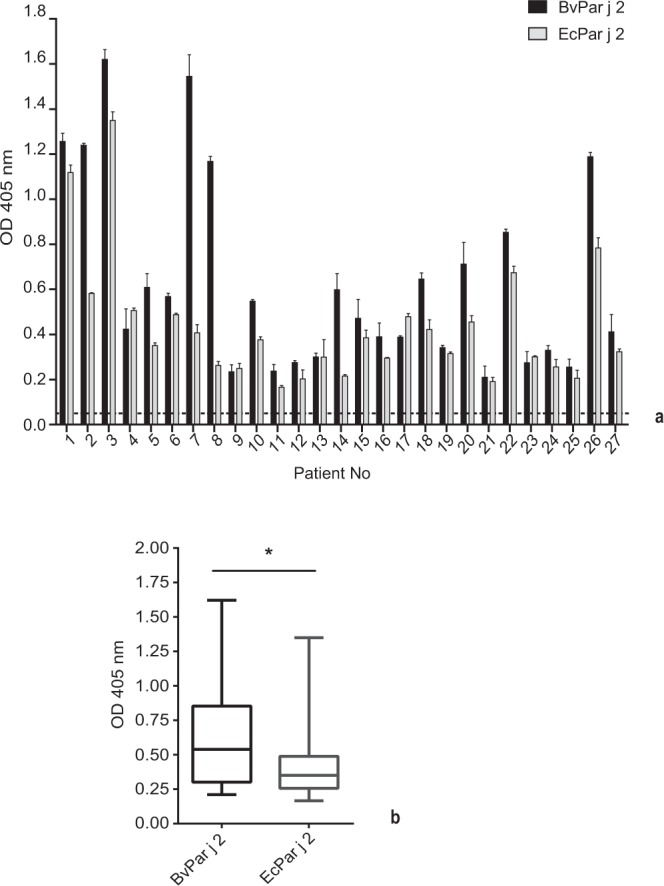
IgE reactivity of BvPar j 2 (black) and EcPar j 2 (grey) demonstrated by ELISA. IgE reactivity (y-axis: mean OD values +/− SD) of sera from 27 *Parietaria* allergic patients (x-axis) to BvPar j 2 and EcPar j 2. The buffer control was subtracted from the data and cut-off is represented by dashed line. **(a)** Box plot representation (whiskers = minimum and maximum; boxes = 25^th^ to 75^th^ percentiles, means: horizontal bars) of the IgE reactivity of the above sera to BvPar j 2 and EcPar j 2. **(b)** Statistically significant differences are indicated (*P < 0.05).

IgE reactivity of BvPar j 2 and EcPar j 2 was compared by testing sera from 27 allergic patients (Supplementary Table S1) (Fig. 4a). Approximately one third of the sera showed higher IgE reactivity to BvPar j 2 as compared to EcPar j 2 whereas the other sera displayed similar IgE reactivity to the two proteins. Serum from a non-allergic subject showed no IgE reactivity. Correct coating of the proteins to the ELISA plates was confirmed with rabbit-anti-Par j 2 antibodies (data not shown). The comparison of the IgE levels specific for BvPar j 2 and EcPar j 2 demonstrated that BvPar j 2-specific IgE levels were significantly higher than EcPar j 2-specific IgE levels (Fig. 4b).

**Figure 5:**
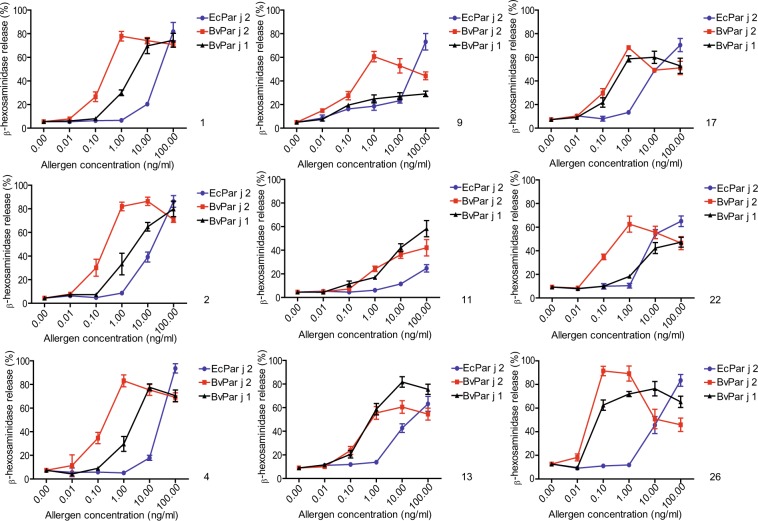
Comparison of the allergenic activity of BvPar j 1, BvPar j 2 and EcPar j 2. RBL cells were loaded with sera from nine *Parietaria* allergic patients (1, 2, 4, 9, 11, 13, 17, 22, 26) and stimulated with different allergen concentrations (x-axes). β-hexosaminidase releases are expressed as percentages of total mediator contents +/− SD (y-axes).

Next we compared the allergenic activity of BvPar j 2, BvPar j 1 and EcPar j 2 in basophil activation experiments. Rat basophil leukemia cells transfected with the human FcεRI receptor were loaded with serum IgE from nine Par j 1 and Par j 2 allergic patients and incubated with 5 different concentrations of each protein (0.01, 0.1, 1, 10, 100 ng/ml) or buffer only. BvPar j 2 was 100–1000-fold more potent in inducing mediator release as compared to EcPar j 2 when comparing the concentrations which induced a comparable release of mediators (Fig. 5). BvPar j 1 was less effective in inducing basophil activation as compared to BvPar j 2 in eight of the nine patients (Fig. 5). Stronger basophil activation with BvPar j 1 compared to BvPar j 2 was noted for two patients (i.e., patients, 11 and 13).

### Par j 2 and Par j 1 show IgE cross-reactivity but represent distinct allergens

**Figure 6:**
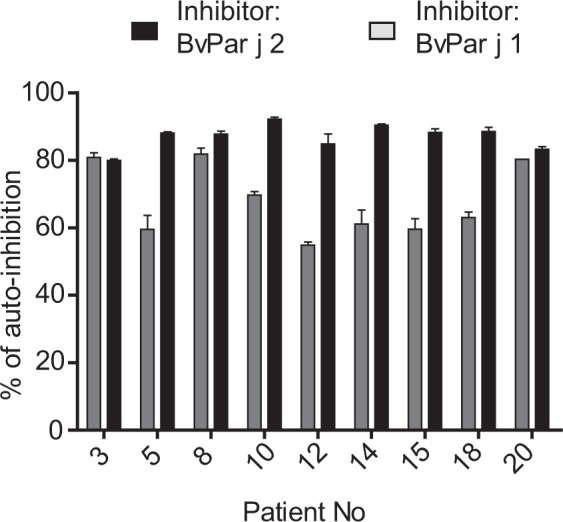
IgE cross-reactivity of Par j 1 and Par j 2 studied by IgE inhibition ELISA. Shown are the percentages of inhibition of IgE binding to BvPar j 1 and BvPar j 2 achieved by pre-incubating sera from patients (x-axis) with the other allergen (Inhibitors: BvPar j 1 grey column; BvPar j 2 black column). Results are expressed as percentages of auto-inhibition +/− SD (y-axis).

IgE cross-reactivity between Par j 1 and Par j 2 was investigated by IgE inhibition ELISA experiments (Fig. 6). Sera from *Parietaria* allergic patients were pre-adsorbed either with Par j 1 (grey) or Par j 2 (black) and then tested for remaining IgE reactivity to the same and to the other allergen (Fig. 6). Figure 6 shows the results of the IgE inhibitions as percentages of auto-inhibition obtained with the same allergen. Par j 2 inhibited IgE binding to Par j 1 better than Par j 1 to Par j 2 in seven of the nine tested patients (i.e., #5, 8, 10, 12, 15, 18). A similar cross-inhibition of IgE binding was found for two of the nine patients (i.e., #3, 20) (Fig. 6). When we calculated the mean percentages of IgE inhibition we found out that Par j 2 inhibited IgE binding to Par j 1 stronger (average 87%) than Par j 1 to Par j 2 (average 68%).

Next we tested rabbit IgG antibodies obtained by immunization with BvPar j 2 for their ability to inhibit allergic patients’ IgE binding to Par j 2 and Par j 1 (Table 1). We found that rabbit anti-BvPar j 2 antibodies inhibited strongly allergic patients IgE binding to Par j 2 (77.7–93.7%; 86.7% mean inhibition) whereas IgE binding to Par j 1 was inhibited to a much lower extent (41.2–73.3%; 56.8% mean inhibition) (Table 1), even for sera which had shown strong cross-reactivity (e.g., patients #3, #20, Fig. 6, Table 1).

**Table 1:** Inhibition of allergic patients IgE binding to Par j 1 and Par j 2 with rabbit anti-Par j 2 antibodies.

No	Par j 1			Par j 2		
	OD		%	OD		%
	non-inhibited	Inhibited		non-inhibited	Inhibited	
#3	1.733	0.744	57	3.5	0.219	93.7
#5	0.698	0.41	41.2	1.5	0.252	83.2
#10	1.144	0.54	52.8	1.216	0.192	84.2
#14	0.694	0.185	73.3	1.23	0.274	77.7
#15	0.481	0.183	61.9	0.886	0.102	88.4
#18	0.705	0.33	53.1	1.414	0.166	88.2
#20	0.904	0.38	57.9	1.575	0.138	91.2
Mean	0.909	0.396	56.8	1.617	0.192	86.7

Shown are optical densities (OD) corresponding to bound IgE antibodies and calculated percentages of IgE inhibition.

### Recombinant Par j 1 and Par j 2 resemble the majority of allergenic IgE epitopes in *Parietaria judaica* pollen

**Figure 7:**
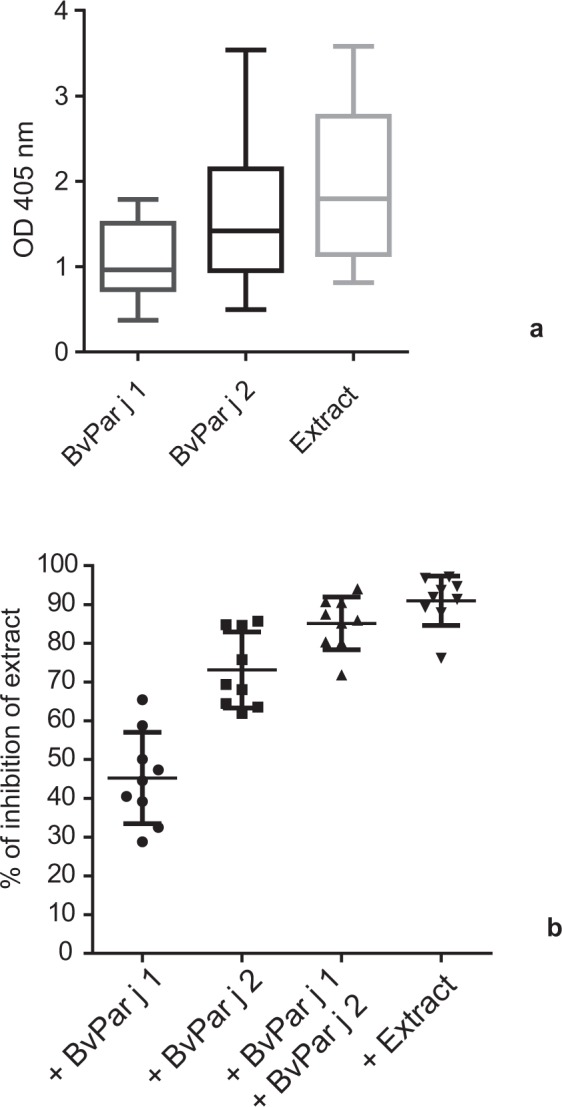
Contribution of Par j 1 and Par j 2-specific IgE to IgE specific for natural *Parietaria* allergens. OD values corresponding to IgE levels specific for BvPar j 1, BvPar j 2 and natural *Parietaria judaica* pollen extract are shown as **(a)** box plot representation (whiskers = minimum and maximum; boxes = 25^th^ to 75^th^ percentiles, means: horizontal bars) (y-axis: OD values). **(b)** Inhibition of IgE binding of sera to *Parietaria judaica* extract by pre-incubation of sera with BvPar j 1, BvPar j 2, with a mix of the two proteins or with extract. Scatter plots showing the percentages of inhibition of IgE binding, horizontal lines indicate means ± SD.

The comparison of IgE levels specific for BvPar j 1, BvPar j 2 and *Parietaria judaica* extract indicates that BvPar j 2-specific IgE levels represent a higher proportion of IgE-specific for natural *Parietaria* allergens than BvPar j 1 (Fig. 7a). Therefore, we were interested to study in detail the contribution of BvPar j 1 and BvPar j 2-specific IgE to IgE specific for natural *Parietaria* allergens. For this purpose, sera from *Parietaria* allergic patients were pre-incubated with BvPar j 1, BvPar j 2, a mix of BvPar j 1 and BvPar j 2 and for control purposes with natural *Parietaria* allergens and remaining IgE binding to natural *Parietaria* allergens was measured to calculate the percentage of inhibition (Fig. 7b). The inhibition of IgE binding to natural *Parietaria* allergens after pre-adsorption with BvPar j 1 ranged from 28.8% to 65.5% (mean 45.2%) and after pre-incubation with BvPar j 2 it ranged from 61.9% to 84.8% (mean 73.1%). The mix of BvPar j 1 and BvPar j 2 inhibited IgE binding to natural *Parietaria* allergens (i.e., 71.9–94.1%; mean 85.1%) almost as good as natural *Parietaria* allergens (i.e., 76.2–97.1%; mean 91%) (Fig. 7b).

## Discussion

The weed wall pellitory, *Parietaria judaica*, is a major allergen source responsible for severe forms of respiratory allergy in the Mediterranean area in Europe as well as in certain parts of USA and Australia. Allergen-specific immunotherapy the only causative and disease-modifying treatment for allergy is based on the vaccination of allergic patients with the disease-causing allergens^24,31^. Allergy vaccines based on natural allergen extracts are difficult to produce in a form which meets the standards of modern vaccines in terms of quality and reproducible production according to good manufacturing practice (GMP) conditions^32^. Accordingly, new types of allergy vaccines based on recombinant allergens, recombinant hypoallergens or allergen-derived peptides are currently being developed and show promising results in clinical trials^26,33,34^. A first crucial step towards the development of a recombinant allergen-based vaccine approach for a given allergen source is the identification of the clinically most important allergen molecules which resemble the epitope spectrum of the allergen source^35^. Our study addresses several important open questions which need to be answered for the preparation of a molecular vaccine approach for the treatment of allergy to *Parietaria judaica*. The first important question is whether a *Parietaria* vaccine should contain only Par j 2 or Par j 1 or both proteins. In fact it has been shown that the major *Parietaria* allergens, Par j 1 and Par j 2 contain cross-reactive IgE epitopes but it has not been studied in detail if IgE cross-reactivity is extensive enough so that one of the two proteins can replace the other^30^. To address this question we have expressed folded versions of Par j 1 and Par j 2 in insect cells. The insect cell-expressed Par j 1 and Par j 2 expressed by us seemed to resemble natural Par j 1 and Par j 2 better than the previously reported recombinant Par j 1 and Par j 2 expressed in yeast because the yeast-expressed proteins were glycosylated whereas the natural proteins were not^20^. Of note, the yeast-expressed Par j 1 displayed even a mixture of two bands of 18.6 and 16.7 kDa. By contrast, our insect cell expressed proteins displayed single bands and resembled the exact molecular weight predicted according to their amino acid sequence without glycosylation. This is important, because it is known that hyperglycosylation as it occurs upon yeast expression can mask IgE epitopes^36^.

We found that recombinant folded Par j 2 had higher IgE binding capacity and allergenic activity than unfolded Par j 2 demonstrating the importance of conformational epitopes in addition to sequential epitopes for IgE reactivity to Par j 2. IgE inhibition experiments performed with Par j 1 and Par j 2 indicated that the allergens contain cross-reactive IgE epitopes but none of the two proteins could completely inhibit IgE binding to the other allergen. These results were also corroborated by the finding that IgG antibodies induced by immunization of rabbits with Par j 2 inhibited allergic patients IgE binding to Par j 2 almost completely but inhibited allergic patients IgE binding to Par j 1 only partially. We therefore concluded that both, Par j 1 and Par j 2 need to be part of a vaccine for allergy to *Parietaria* which is in contrast to a previously published studies concluding that Par j 2 is sufficient for diagnosis and allergen-specific immunotherapy (AIT)^16,20,37^.

Our conclusion, that both, Par j 1 and Par j 2 are needed for AIT of *Parietaria* allergy, was confirmed by two other observations: The comparison of the allergenic activity of Par j 1 and Par j 2 in basophil activation tests showed that both proteins are highly allergenic and induced strong basophil degranulation *in vitro* which is a good surrogate marker for the *in vivo* allergenic activity of an allergen^38,39^.

Importantly, we found different types of *Parietaria* allergic patients, one type, i.e., the majority of patients who were more sensitive to Par j 2 but also another type of patients who reacted stronger to Par j 1.

Finally, yet another important question remained to be answered: Do Par j 1 and Par j 2 resemble the majority of IgE epitopes of a natural *Parietaria* allergen extract or are other allergens needed? In fact, profilins and calcium-binding allergens have been described as cross-reactive allergens in *Parietaria* which are recognized by approximately 10% of the patients^18,19^. To address the latter question we performed IgE inhibition studies which demonstrated that a mix of Par j 1 and Par j 2 almost completely inhibited IgE binding to *Parietaria* extract and thus resembled the IgE epitopes of a complete natural *Parietaria* allergen extract. Of note, the mixture of the insect cell-expressed rPar j 1 and rPar j 2 reported by us seemed to inhibit IgE binding to *Parietaria* pollen extract better (mean inhibition 85.1% which was almost as good as natural *Parietaria* allergens mean inhibition 91%) than the previously reported rPar j 1 and rPar j 2 expressed in yeast^20^.

Our study thus identifies Par j 1 and Par j 2 as the two allergen molecules which need to be included in a molecular vaccine for the treatment of *Parietaria* allergy. In fact, the two recombinant folded versions of Par j 1 and Par j 2 could be formulated as allergy vaccine containing wildtype-like recombinant allergens as has been shown for a birch pollen allergy vaccine based on recombinant wildtype-like major birch pollen allergen Bet v 1 which was effective in clinical trials^40,41^. Likewise it was shown that a mix of the major recombinant grass pollen allergens, Phl p 1, Phl p 2, Phl p 5 and Phl p 6 which were part of a recombinant allergen-based grass pollen vaccine was effective to treat grass pollen allergy^42^. Accordingly one may consider formulating a recombinant wild-type allergen vaccine based on Par j 1 and Par j 2 for the treatment of *Parietaria* allergy. However, the recombinant wild-type-like folded Par j 1 and Par j 2 molecules may be used also as fully allergenic benchmarks for the construction of vaccines based on recombinant hypoallergenic derivatives of Par j 1 and Par j 2 as described for the recently developed hypoallergenic grass pollen allergy vaccine, BM32^43–45^.

## Materials and Methods

### *Paritaria judaica* pollen extract

An allergen extract was prepared from *Parietaria judaica* pollen which was obtained from ALK-Abelló (Madrid, Spain). An aqueous extract was prepared by stirring 1 g of pollen in physiologic buffer (PBS, pH = 7.4) during 4 hours at RT followed by two rounds of centrifugation at 15.000xg for 15 min to remove insoluble particles. The supernatant was dialyzed against dH_2_O overnight at 4 °C and stored in aliquots at −20 °C. The protein concentration of the dialysed samples was determined using a Bradford protein assay kit (Bio-Rad Laboratories, Hercules, CA).

### Recombinant production of Par j 2 and Par j 1 in baculovirus-infected insect cells (BvPar j 2 and BvPar j 1)

#### Cloning and generating of high molecular weight recombinant bacmid DNA

Synthetic genes, codon-optimized for expression in insect cells coding for Par j 2.0101 (340 base pairs; accession number P55958) and for Par j 1.0102 (451 base pairs; accession number O04404) both including an additional 3′ sequence coding for a C-terminal hexahisitidine tag (Table 1), were subcloned into plasmid pTM1 via the BamHI/SmaI sites (ATG: biosynthetics, Merzhausen, Germany). The plasmids were transformed into DH10Bac^tm^ *E. coli* cells to generate high molecular weight recombinant bacmid DNA which was then used to transfect Sf9 insect cells to obtain virus stocks^46^.

#### Expression and purification of recombinant BvPar j 2, BvPar j 1 and EcPar j 2

For expression of the recombinant BvPar j 2 and BvPar j 1 proteins 150 μl/ml of viral stock was added to 20 ml of 1 × 10^6^ cells/ml of SF9 cells in culture medium (Sf-900 II SFM, Gibco, Life technologies, Carlsbad, CA) supplemented only with gentamicin (10 μg/ml, Life technologies) but without FBS. The cells were then incubated under continuous shaking (120 rpm) for 48 hours at 27 °C. Cells and supernatant were then separated by centrifugation (1000 rpm, 10 min, 4 °C) and the supernatant was dialysed against buffer A used for purification by Ni^2+^-affinity chromatography (50 mM NaH_2_PO_4_, 300 mM NaCl, 10 mM Imidazole, pH 8.0) at 4 °C. Affinity purification of recombinant proteins was achieved by using Ni-agarose (Qiagen, Hilden, Germany)^46^.

Bacterially-expressed recombinant Par j 2 was expressed and purified as described and received from Prof. Paolo Colombo^12^.

The purity of the recombinant proteins was assessed by SDS-PAGE under non-reducing and reducing conditions with either ß-Mercaptoethanol or Tris(2-carboxyethyl)phosphine (TCEP) (see Supplementary Fig. S2). Aliquots of 2 μg of BvPar j 1, BvPar j 2 and EcPar j 2 were loaded on a 14% analytical gel^47^. Pre-stained protein ladder (PageRulerTMPlus, Thermo Scientific) was used as a molecular weight marker to determine the apparent masses of the proteins after staining of the gels with Coomassie blue.

#### Biochemical and biophysical characterization of purified BvPar j 1, BvPar j 2 and EcPar j 2

The determination of the mass of the purified proteins was performed by means of MALDI-ToF MS as previously described^48^. The fold of the proteins was analyzed by circular dichroism (CD) measurements as described^49^. Aggregation behaviour of purified proteins was analysed by gel filtration on an UltiMate™ 3000 HPLC (Dionex-Thermo Fisher Scientific, Vienna, Austria) using a BioSep-SEC-s3000 column (Phenomenex, Torrance, CA). Gel filtration of recombinant proteins was done in PBS pH 7.5. Cytochrome C (12.4 kDa) (Sigma-Aldrich, St. Louis, Mi) and carbonic anhydrase (29 kDa) (Sigma-Aldrich) were used for calibration under identical conditions and the molecular masses (MMs) of the elution peaks were calculated based on the elution time of the standard proteins^49^.

#### Sera from *Paritaria judaica*﻿ allergic patients

Sera from patients (n = 27; age 7 to 62 years; 19 females and 8 males) suffering from symptoms of pollinosis during the flowering period of *Parietaria* were obtained in the Associated Centers for Molecular Allergology, Rome, Italy. Clinical symptoms were recorded, IgE sensitization to *Parietaria* was confirmed by the measurement of Par j 2-specific IgE Abs (ImmunoCAP ISAC, Phadia, Uppsala, Sweden) and IgE sensitizations to other allergen sources were determined by ImmunoCAP ISAC measurements of IgE specific for micro-arrayed allergens (see Supplementary Table S1).

Serum samples were analyzed in an anonymized manner with approval of the ethics committee of the Medical University of Vienna, Austria (EK1641/2014). All experiments were performed in accordance with relevant guidelines and regulations.

#### IgE reactivity and allergenic activity of purified BvPar j 1, BvPar j 2 and EcPar j 2

The reactivity of recombinant allergens with patients IgE was tested by ELISA. Nickel pre-coated ELISA 96-well plates (Thermo Fisher Scientific, Pierce, Rockford, IL) with specific binding capacity for His-tagged proteins were coated with 2 μg/mL of EcPar j 2, 2 μg/mL of BvPar j 2 and, for control purposes, with 2 μg/mL BSA in carbonate buffer for 1 hour RT. In pilot experiments we tested different dilutions of sera to assure conditions of allergen excess. The plates were then washed 3 times with PBST (PBS + 0.05% Tween 20) and incubated overnight with sera (diluted 1:10 PBST + BSA) from allergic patients, serum from 3 non-allergic subjects. Bound IgE antibodies were detected with horseradish peroxidase–coupled goat anti-human IgE antibodies (KPL, Gaithersburg, MD) diluted 1:2500. The colour reaction was started by adding 1.7 mM 2.2′-azino-bis (3-ethylbenzothiazoline-6-sulfonic acid, Sigma-Aldrich), in 60 mM citric acid, 77 mM Na_2_HPO_4_.2H_2_O, and 3 mM H_2_O_2_. Optical density values (OD) were measured on ELISA reader (Spectra Max PLUS 384, Molecular Devices, Biberach, Germany) at different time points. All determinations were conducted as triplicates and results are displayed as means +/−SD. The buffer control was subtracted from the data and cut-off values of IgE reactivity were defined as mean OD plus three standard deviations of three sera from non-*Parietaria*-sensitized individuals.

To analyze the allergenic activity of the recombinant allergens rat basophil leukemia (RBL) cell-release assays were performed. Rat basophil leukemia (RBL) cells (clone RBL-703/21)^50^ transfected with human FcεRI (triplicates) were loaded with serum samples of nine *Parietaria*-allergic patients diluted 1:10 and incubated overnight at 37 °C. Degranulation of the cells was induced by adding BvPar j 1, BvPar j 2, EcPar j 2 (100, 10, 1, 0.1, 0.01 ng/mL) or buffer as a control. The release of β -hexosaminidase was measured and the results are shown as the percentage of total β-hexosaminidase release^51^.

#### Testing for IgE cross-reactivity by ELISA inhibition experiments

For IgE ELISA competition experiments, 96-well plates (Nunc Maxisorb, Roskilde, Denmark) were coated with BvPar j 1 or BvPar j 2 at a concentration 1 μg/mL or, in certain experiments with *Parietaria judaica* extract in a concentration of 5 μg/ml, and blocked as described for the IgE ELISA. Serum samples from *Parietaria*-allergic patients and from a non-allergic subject (1:20 dilution) were pre-incubated with purified recombinant allergens (BvPar j 2 or BvPar j 1) at a concentration of 2 μg/mL, with *Parietaria judaica* extract (10 μg/ml) or, for control purposes, with BSA (2 μg/ml). Plates were then incubated with pre-adsorbed sera overnight at 4 °C. and bound IgE was detected with horseradish peroxidase–coupled goat anti-human IgE antibodies (KPL, Gaithersburg, MD) diluted 1:2000 as described for the IgE ELISA. All determinations were conducted as duplicates with a variation of less than 5%. The percentages of inhibition obtained by pre-incubating sera with different allergens and allergen extract were calculated as follows: Percentage inhibition = 100 − (mean OD inhibited with allergen/mean OD non-inhibited) × 100. OD non-inhibited corresponds to pre-incubation with BSA as control protein. For certain experiments the percentage of inhibition obtained with another allergen is expressed as percentage of inhibition obtain with the very same allergen (i.e., auto-inhibition).

#### Inhibition of patients IgE binding with specific IgG antibodies obtained by immunizing rabbits with BvPar j 2

Par j 2-specific rabbit antibodies were obtained by immunizing a New Zealand white rabbit three times with the purified protein (200 μg, using once Freund’s complete and twice Freund’s incomplete adjuvant) by Charles River (Chatillon, sur Chalaronne, France). The ability of Par j 2-specific rabbit IgG to inhibit allergic patients IgE binding to Par j 1 and Par j 2 was determined by using an IgE competition ELISA^52^. The concentrations of BvPar j 1 and BvPar j 2 used for coating were 1 μg/mL in these experiments. The rabbit anti-Par j 2 antiserum was diluted 1:50 and sera from allergic patients were diluted 1:20 for IgE detection. All determinations were carried out in duplicates with a variation of less than 5%, results are expressed as means. Percentages of inhibition of IgE binding were calculated as follows: Percentage inhibition = 100 – (OD rabbit immune serum/OD rabbit pre-immune serum) × 100.

#### Statistical analysis and prediction tools

Statistical analysis was performed using GraphPad Prism 6 Software. Mann-Whitney U-test was used for comparison of differences between the groups. All calculated probability (p) values were two-tailed, and differences were considered as statistically significant if the p-value was lower than 0.05. For sequence alignment Clustal Omega program was used (https://www.ebi.ac.uk/Tools/msa/clustalo/). Sequences of LTPs with homology to Par j 2 were identified by comparing the amino acid sequences of Par j 2 and Par j 1 with the sequences deposited in the UniProt data base (www.uniprot.org/blast/) using the BLAST P program.

A hydrophobicity plot of Par j 1 and Par j 2 was generated using the ProtScale bioinformatics tool from the ExPASY server^53^ (http://web.expasy.org/protscale/) using the algorithm of Kyte and Doolittle^54^.

Phylogenetic comparisons of the amino acid sequence of Par j 2 with LTPs from different species were performed using the molecular evolutionary genetics analysis program (MEGA) version 7.0.26^55,56^. We used Neighbor-Joining algorithm with 100 replicate bootstraps^57^.

## Supplementary information

Data set 1

## Data Availability

The datasets generated during and/or analyzed during the current study are available from the corresponding author on reasonable request.
