# Supplementary material for: Expression and characterization of recombinant Par j 1 and Par j 2 resembling the allergenic epitopes of Parietaria judaica pollen
Source: Sci Rep. 2019 Oct 21;9:15043. doi: 10.1038/s41598-019-50854-1 (PMC6803649; doi:10.1038/s41598-019-50854-1)
Supplement: Supplementary file 1 — Data set 1 [file 41598_2019_50854_MOESM1_ESM.doc]

**Expression and characterization of recombinant Par j 1 and Par j 2 resembling the allergenic epitopes of *Parietaria judaica* pollen**

*Yulia Dorofeeva^1^, Paolo Colombo^2^, Miguel Blanca^3^, Adriano Mari^4^, Roman Khanferyan^5,6^, Rudolf Valenta^1,7,8^, Margarete Focke-Tejkl^1^**

^1^Division of Immunopathology, Department of Pathophysiology and Allergy Research, Center of Pathophysiology, Infectiology and Immunology, Medical University of Vienna, Vienna, Austria, ^2^Istituto di Biomedicina ed Immunologia Molecolare “Alberto Monroy” del Consiglio Nazionale delle Ricerche, Palermo, Italy, ^3^Hospital Civil, Malaga, Spain, ^4^Associated Centers for Molecular Allergology, Rome, Italy, ^5^People´s Friendship University, Moscow, Russian Federation, ^6^Scientific and Clinical Center of Otorhinolaryngology of the Federal Medico-Biological Agency, Moscow, Russian Federation, ^7^The Institute of Immunology, Moscow, Russian Federation, ^8^Laboratory for Immunopathology, Department of Clinical Immunology and Allergy, Sechenov First Moscow State Medical University, Moscow, Russian Federation.

**Supplementary Figures and Tables**

**Supplementary Figure S1.**(**a**) Percentages of amino acid sequence identities between Par j 2 and Par j 1 and allergenic lipid transfer proteins (LTP) from other plants. Colored are identities of 60-70% (yellow), >70-80% (orange) and >80% (red). (**b**) Phylogenetic tree of the LTP sequences from (**a**).

**Supplementary Figure S2.** Coomassie brilliant blue-stained SDS-PAGE containing purified BvPar j 1, BvPar j 2 and EcPar j 2 separated under non-reducing conditions (panel NR), reducing conditions using β-Mercaptoethanol (panel Me) or Tris(2-carboxyethyl)phosphine (panel TCEP). Molecular weight markers are indicated in kDa (M).

**Supplementary Table S1.** Demographic, clinical and serological characterization of tested individuals

f, female; m, male; y, years; nd, not detected; ISU-IgE, ISAC standardized units for IgE; ISAC, immuno-solid phase allergen chip; R: Rhinitis; C: Conjunctivitis; AS: Asthma.

**Supplementary Figure S1**

**a**

**b**

**Supplementary Figure S2**

BSA

**Supplementary Table S1.**  Demographic, clinical and serological characterization.

|  | **No** | **Sex (m/f)** | **Age**  **(x-y**  **/mean)** | **Symptoms induced by *Parietaria* pollen** | | | **Par j 2 (ISU)**  **x-y/mean** | **Other pollen**  **allergens** | **HDM allergy** | **Animal allergy** | **Food allergy** |
| --- | --- | --- | --- | --- | --- | --- | --- | --- | --- | --- | --- |
|  |  |  |  | **R** | **C** | **AS** |  |  |  |  |  |
| **Allergic patients** | n=27 | 8 / 19 | 7-62 / 32.4 | n=27 | n=27 | n=27 | 11.4-117.11 / 38.99 | n=24 | n=12 | n=11 | n=11 |
| **Non-allergic**  **controls** | n=3 | 1/2 | 27-55 / 38.3 | n=0 | n=0 | n=0 | nd | n=0 | n=0 | n=0 | n=0 |

Abbreviations: f, female; m, male; y, years; nd, not detected; ISU, ISAC standardized units for IgE; R: Rhinitis; C: Conjunctivitis; AS: Asthma.
